# Yearlong analysis of bacterial diversity in hospital sink drains: culturomics, antibiotic resistance and implications for infection control

**DOI:** 10.3389/fmicb.2024.1501170

**Published:** 2025-02-14

**Authors:** José Laço, Sergi Martorell, Maria del Carmen Gallegos, Margarita Gomila

**Affiliations:** ^1^Microbiology Laboratory (Biology Department), University of the Balearic Islands, Palma, Spain; ^2^Microbiology Unit, Hospital Son Llàtzer, Palma, Spain

**Keywords:** bacterial diversity, culturomics, antibiotic resistance, sink drains, *Pseudomonas aeruginosa*, infection control

## Abstract

Hospitals can carry high levels of bacterial diversity from all types of origins, such as human skin, outside environment and medical equipment. Sink drains in clinical settings are considered reservoirs for pathogenic bacteria and potential sources of hospital-acquired infections (HAI’s) and antibiotic resistance genes (ARGs). Therefore, infection control measures are crucial to minimizing the risks associated with these reservoirs. Recent research has focused primarily on intensive care units (ICUs) and known pathogens, often employing metagenomic approaches that do not include bacterial isolation. This study aims to evaluate bacterial diversity using culturomics, extending the investigation beyond the ICU to identify antibiotic-resistant bacteria. A total of four samplings were conducted over 1 year (March 2022 to March 2023) in five different hospital wards [ICU, General Medicine (GM), Hematology (H), Short stay unit (UCE), and Microbiology laboratory (MS)]. All samples were cultured on selective and non-selective culture media, resulting in 1,058 isolates identified using MALDI-TOF MS, with a subset confirmed through 16S rRNA gene sequencing. Isolates retrieved from antibiotic supplemented agar were subjected to antibiotic susceptibility testing. The highest bacterial diversity, as measured by the Shannon index, was observed in the ICU and GM wards, posing significant risks to patients in these areas. While bacterial genera were largely similar across wards and sampling times, with *Pseudomonas* and *Stenotrophomonas* being the most prevalent, different species were detected in each sampling, indicating no loss of diversity. This suggests that these environments undergo dynamic changes over time, influenced by their surroundings. The results also indicate a relationship between human activity and drain usage and the presence of *Pseudomonas aeruginosa*, the most commonly found species across most wards. Antibiotic susceptibility testing revealed that all tested isolates, except for one, were multi-resistant, including clinically relevant species, such as *P. aeruginosa* and *K. pneumoniae*. Hospital drains may serve as reservoirs for both known and emerging pathogens exhibiting high antibiotic resistance phenotypes. Their dynamic nature may provide insights into strategies for preventing the colonization of these environments by such species.

## 1 Introduction

Hospital environments can carry a significant level of microbial diversity, leading to the development of complex microbial communities (Lax et al., 2017; Oberauner et al., 2013). Bacteria forming the microbiome in these environments can have a multitude of origins, including human skin, from both patients and workers, air from the outside environment or medical equipment (Rintala et al., 2008). Another critical factor influencing the clinical microbiome is the environmental conditions, which affect the selection of certain microorganisms (Martiny et al., 2006). Variables such as air temperature and relative humidity, patient flow, ventilation, and cleaning practices all contribute to the prevalence of potential pathogenic bacteria within hospital settings (Hassan and Zeeshan, 2022).

Within hospitals, many surfaces and areas may serve as bacterial reservoirs, with some locations or settings being particularly significant for understanding infection dynamics. The location is also crucial, in fact several authors report that intensive care units (ICU) are especially vulnerable to problems arising from bacterial reservoirs (Maseda et al., 2014).

Sink drains are well-known reservoirs for diverse bacterial communities (Kizny Gordon et al., 2017), potentially harboring aerosolized pathogens or opportunistic microorganisms (Bloomfield et al., 2015). These environments may not only contain clinically relevant bacteria but also serve as reservoirs for mobile genetic elements carrying antibiotic resistance or virulence genes. In addition, given the diverse sources of bacteria in drains, they may also serve as indicators of species present throughout the hospital, and potentially as sources of infection (Parkes and Hota, 2018).

The bacterial population colonizing these environments, range from commensals to opportunistic pathogens, including common nosocomial infection agents and antibiotic-resistant bacteria (Oberauner et al., 2013). Nosocomial infections, or hospital acquired infections (HAIs), are increasingly problematic worldwide, causing higher mortality and morbidity in patients, and imposing a significant economic burden on healthcare systems, potentially consuming up to 6% of public hospital budgets (Slawomirski et al., 2017). In the European Union and European Economic Area (EU/EEA), more than 3.5 million cases of HAIs are estimated to occur annually, resulting in more than 90,000 deaths and corresponding to approximately 2.5 million disability-adjusted life years (DALYs) (Cassini et al., 2016). HAIs are also the sixth leading cause of death in the United States, corresponding to 99,000 annual deaths (Liu and Dickter, 2020).

Selective pressures within hospital environments, particularly those arising from antibiotic use, significantly influence the resident microbiota, increasing levels of resistance. Antibiotic selection pressure is especially concerning when resistance genes are associated with mobile genetic elements, such as plasmids, which facilitate their dissemination among microorganisms. Thus, hospital environments not only act as reservoirs for bacteria capable of triggering an infection but also as the perfect environments for the dissemination or emergence of new antibiotics resistances or virulence mechanisms. Potentially infectious agents present in the environment can enter hospitals through various routes, including water supplies and distribution systems. Bacteria can colonize these systems through biofilm formation, making them substantial reservoirs for various bacteria, including potential pathogens and/or carriers of antibiotic resistance genes (ARG) (Cantón et al., 2013; Pettigrew et al., 2016).

While many studies focus on the presence and abundance of well-known pathogenic strains, such as those “ESKAPE” group, *Enterococcus* spp., *Staphylococcus* spp., *Klebsiella* spp., *Acinetobacter* spp., *Pseudomonas aeruginosa*, and *Enterobacter* spp. (Denissen et al., 2022; Rizki et al., 2024), other potentially harmful bacteria may also inhabit these environments, posing risks, especially to immunocompromised patients. These include species such as *Mycobacterium llatzerense* (Cárdenas et al., 2014), *Pseudomonas putida* (Fernández et al., 2015), *Brucella anthropii* (Wang et al., 2016) or the emerging pathogen *Stenotrophomonas maltophilia* (Looney et al., 2009). These bacteria are often overlooked in studies focused solely on known pathogens, despite their potential to cause harm in specific settings.

While the ICU is the most studied area for HAIs, other hospital wards are also important and need careful attention due to their high patient turnover, including immunocompromised individuals, even outside major risk areas like the ICU (Freixas et al., 2013; Rhee et al., 2015; Sopena and Sabrià, 2005). Therefore, it is crucial to study various hospital environments, to understand the diversity that they harbor and evaluate the potential risks associated with their presence.

Understanding the microbial composition in hospital environments is essential for effective infection control. While recent studies have focused on microbiome analysis using culture-independent methods, heavily relying on metagenomic approaches (Lax et al., 2017; Shobo et al., 2020), these approaches often lack bacterial isolation and precise species identification, hindering more in-depth investigations (Cronquist et al., 2012; Lagier et al., 2015).

Our study aims to address a crucial gap in understanding the dynamics of bacterial colonization in hospital environments over the course of a one-year study. By conducting a comprehensive analysis of bacterial diversity in sink drains across various hospital wards (Intensive Care Unit, Hematology, Short Stay Unit, General Medicine, and the Microbiology laboratory) we seek to uncover valuable insights into the role of sink drains as a reservoir for potential pathogens and antibiotic resistance, with special emphasis on carbapenemase-producing bacteria. This knowledge is essential for the development and implementation of targeted infection control measures, ultimately reducing the risk of healthcare-associated infections.

## 2 Materials and methods

### 2.1 Sample collection

A total of four samplings were conducted over the course of 1 year (February 2022, June 2022, September 2022 and February 2023) across five different hospital wards in a hospital in Mallorca: The Intensive Care Unit (ICU), Hematology (H), Short Stay Unit (UCE), General Medicine (GM) and Microbiology Laboratory (MS). Additionally, during the final sampling, a newly established ICU unit was included (ICU_New).

The MS sink was routinely cleaned with bleach, while the others were cleaned daily by the cleaning staff following their cleaning protocols. In addition to routine cleaning, established disinfection procedures were applied throughout the hospital. Drains were disinfected with a chemical disinfectant followed by steam under pressure every 15 days in patient areas and monthly in non-patient areas. Pipes were hyperchlorinated at a low temperature annually.

Sampling locations varied by ward. In the ICU and MS samples were collected in drains from common areas constantly in use, with the exception of the ICU drain collected in D6 sampling, which was used more sporadically at that time. Samples from the UCE and GM wards were taken from drains in patient rooms, normally occupied, while samples from the hematology ward (H) were collected from drains in patient rooms that were sporadically occupied.

Swabs were used to collect samples from the drains in each ward. Once collected in a Ringer solution of 4–5 mL, the samples were promptly transported to the laboratory and processed within 2 h. For culturing, 100 μL of each dilution was plated in duplicate on five different culture media: non-selective media include R2A agar (Scharlab, Spain) and chocolate agar (Biomerieux, France), and selective media with antibiotic pressure, including ChromID^®^ ESBL agar (Biomerieux, France) and ChromID^®^ CarbaSmart Agar (Biomerieux, France), as well as Cetrimide agar (Merck, Germany), selective for *Pseudomonas* spp.

### 2.2 Plate counts and isolation

**Figure 1:**
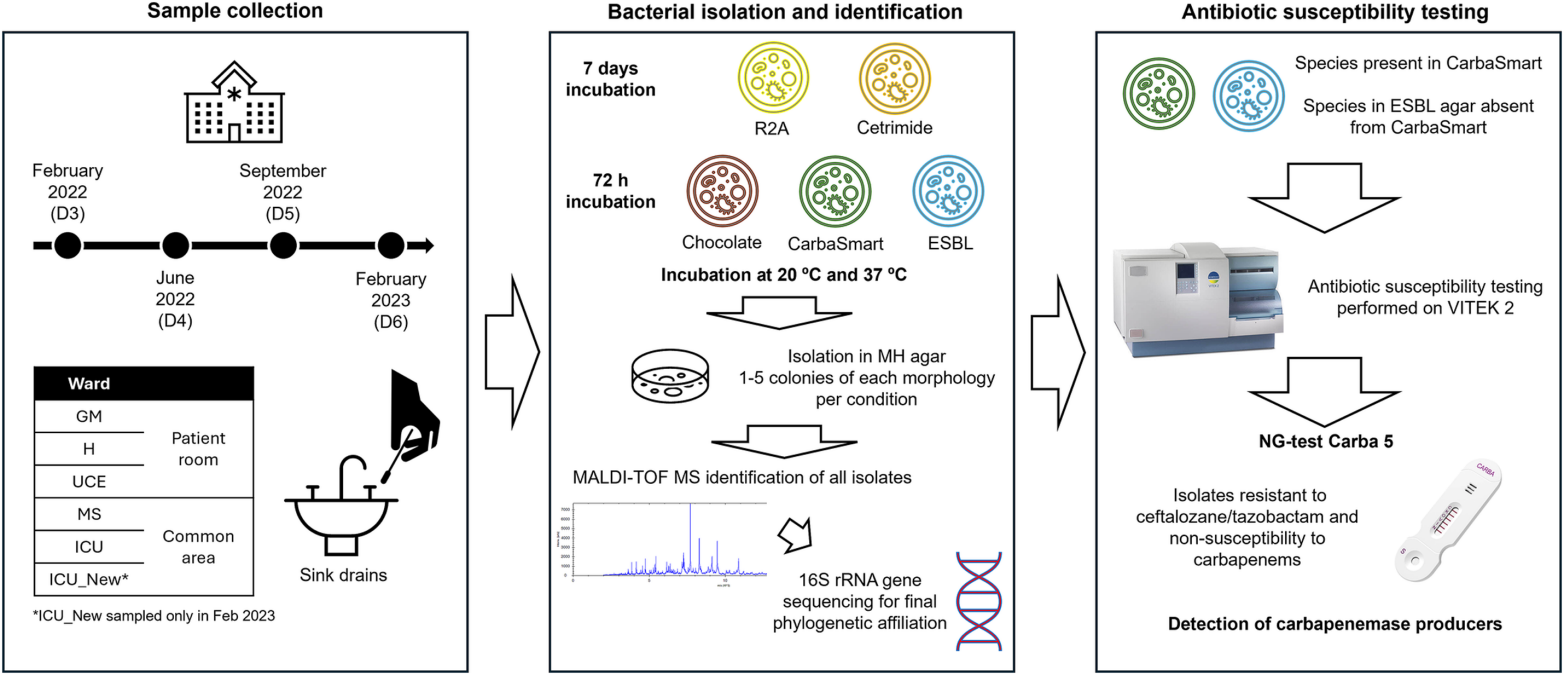
Schematic image representing the experimental design followed.

Incubation was performed for 72 h on ChromID^®^ ESBL, ChromID^®^ CarbaSmart and Chocolate agar, and for 7 days in R2A and Cetrimide agar. Incubation temperatures were set at both 20°C and 37°C. Following incubation, plates containing between 30 and 300 colonies for each culture media were counted and colony-forming units per milliliter (cfu/mL) were calculated. One to five colonies of each observed morphology in each plate were selected and individually isolated on Muller-Hinton agar (Condalab, Spain), followed by a second culture to assure isolation of independent colonies. These isolated colonies were then incubated at 20°C or 37°C, depending on the original growth temperature, for 24–48 h to facilitate further characterization and identification. All isolates were stored in salts −70 and kept at −80°C. Figure 1 presents a schematic illustration of the experimental methodology followed.

### 2.3 Identification of isolates

#### 2.3.1 MALDI-TOF MS identification

Isolates were subjected to matrix-assisted laser desorption/ionization time of flight mass spectrometry (MALDI-TOF MS) analysis for initial identification, following the protocol described by Bruker Daltonik for protein extracts. An extraction was made by suspending a loop of a fresh bacterial colony in 300 μL milliQ water and mixed with 900 μL of absolute ethanol. The mixture was then centrifuged at 13,000 rpm for 2 min. After decanting the supernatant, the pellet was airdried briefly, for a few minutes, before being resuspended in an equal volume of 70% formic acid and 100% acetonitrile by pipetting. Finally, it was centrifuged at 13,000 rpm for 2 min and the resulting supernatant was stored in a new tube at −80°C until use. For the MALDI-TOF MS analysis, the final product was applied in triplicate to a 96-spot polished steel target plate (Bruker Daltonik, Germany), dried at room temperature, and overlaid with 1 μL of 10 mg/mL alpha-4-cyano-4-hydroxycinnamic acid (CHCA, Bruker Daltonik, Germany) dissolved in 50% acetonitrile and 2.5% trifluoroacetic acid and allowed to dry. Approximately 500 shots were collected for each spectrum, and a final identification score was generated. Identification scores were categorized as follows: unreliable identification (score 0–1.69), low-confidence species identification (score 1.7–1.99) and high-confidence species identification (score 2.00–3.00). *Escherichia coli* DH5α was used as a quality control. Bacterial groups were established based on protein profile similarity and the identification scores obtained.

Dendrograms were constructed for each sampling utilizing the MALDI-TOF MS spectra results of individual isolates, employing the MALDI Biotyper OC, version 3.1.66 program (Bruker Daltonik GmbH). Groups were established based on branch groupings and scores obtained in the species identification.

#### 2.3.2 16S rRNA gene amplification and sequencing

To confirm phylogenetic affiliations, some isolates from each of those established groups were selected for amplification and sequencing of the 16S rRNA gene. DNA extraction was performed using the Wizard Genomic DNA Purification kit (Promega, Madison, WI, United States). PCR amplifications were performed using the primers described by Lane (1991). PCR program was as follows: a starting step of incubation at 94°C for 5 min, followed by 30 cycles of denaturation at 94°C for 1 min, annealing at 55°C for 1 min, elongation at 72°C for 2 min, and a final elongation step at 72°C for 10 min. The PCR products were purified with MultiScreen HTS PCR 96-well filter plates (Millipore) and prepared for Sanger sequencing by adding 1 μL of 5 mM primer to 10 μL of purified product. Sanger sequencing was performed to Secugen (Madrid, Spain). Final identification for each isolate was refined by comparing the initial MALDI-TOF MS identification with the 16S rRNA gene sequence data. In cases where species identification was unclear, the genus name followed of species acronym, sp., was utilized. A dendrogram with all the isolates sequenced was also generated for a more precise phylogenetic assignation.

### 2.4 Alfa and beta diversity of the bacterial communities in each ward

Diversity indexes were calculated for each sampling in each individual ward based on the counts of species/phylogenetic taxa defined, using the PAST 4.14 program. Alfa-diversity was assessed using box plots for Shannon (H), Simpson (λ) Richness (S) and Chao-1 indices. Beta-diversity was evaluated through community ordination using Principal Coordinate Analysis (PCoA) based on the Bray-Curtis index, to observe distances between samples.

### 2.5 Antibiotic susceptibility testing and carbapenemase screening

A selection of isolates obtained from antibiotic-supplemented media was subjected to antibiotic susceptibility testing using the VITEK 2 system. These isolates were selected based on growth in the ChromID^®^ CarbaSmart Agar, except for *Stenotrophomonas* spp., and isolates from species not represented in that agar, but present on ChromID^®^ ESBL agar. After overnight incubation at 30°C in Muller Hinton agar, 2–3 colonies from pure cultures were resuspended in 0.45% NaCl solution to achieve a suspension of 0.5 McFarland scale standard, and VITEK 2 susceptibility testing was performed according to the manufacturer’s instructions using the appropriate cards: AST-N425 for *Enterobacteriaceae* and AST-N427 for *Pseudomonas* species and other Gram-negative non-fermentative bacteria. Antibiotic resistance phenotypes were automatically reported through VITEK 2 AES software 05.01. Bacteria were considered multi-resistant when they were non-susceptible to antibiotics included in three or more classes (Magiorakos et al., 2012). In addition, isolates that exhibited resistance to the combination of ceftolozane/tazobactam, along with non-susceptibility to carbapenems, were screened for the presence of the five most common carbapenemase families (KPC, OXA-48-like, VIM, IMP, and NDM) using the NG-test Carba 5 (NG-Biotech, France). Moreover, isolates that did not yield a result from the VITEK2 were also included in this part of the study. For this test, 3–4 colonies were inoculated into a 1.5-mL microcentrifuge tube containing 5 drops of extraction buffer, and vortexed for approximately 10 s. One hundred microliter of the suspension was inoculated into the NG-Test Carba 5 sample well. After 15 min, the test was visually examined for the presence or absence of the control and test lines.

### 2.6 Statistical analysis

All plots were generated using RStudio with R version 4.3.3, utilizing the ggplot2, readxl, tidyverse, vegan, patchwork and RColorBrewer packages. Statistical analysis of the Shannon index values was performed using an ANOVA test, and beta diversity index was analyzed using PERMANOVA.

## 3 Results

### 3.1 Plate counts and isolates retrieval

CFU/mL were calculated for sink drain samples from all wards (Supplementary Table S1). The highest values were observed on R2A agar, while the lowest values were found on antibiotic-supplemented media, with values ranging from 10^1^ to 10^6^ CFU/mL. Plates incubated at room temperature generally yielded the highest counts. Additionally, in some cases, growth was observed at room temperature but not at 37°C, such as D3 for ICU, and UCE, D4 for UCE and H and D5 for ICU and MS. Conversely, growth was registered at 37°C but not at room temperature for ICU D5 sample on R2A agar. Overall, the range of values at room temperature was 10^1^ to 10^5^ CFU/mL compared to growth at 37°C.

A total of 1,058 isolates were obtained, with the ICU and GM wards registering the highest number, and the MS service with the lowest counts. In some samplings, a lower number of isolates were retrieved due to overestimation of dilutions based on expected results from previous samplings, particularly in D5 for ICU and D6 for GM.

### 3.2 Characterization and identification of isolates

All isolates were initially identified using MALDI-TOF MS. High-confidence species-level identification (score > 2.0) was obtained for 802 isolates (76% of the total), while 173 isolates (16% of total) were identified at the genus level. The remaining 8% had unreliable identifications. Dendrograms for each sampling were generated to compare the protein profiles within strains (data not shown). A selection of isolates was selected for 16S rRNA sequencing allowing to confirm the previous identification and clarify taxonomic affiliation of unreliable identification isolates. To compare the species affiliation between samplings a dendrogram with all the isolates sequenced was also generated allowing a more precise phylogenetic assignation (data not shown). All identifications are depicted in Supplementary Table S2.

### 3.3 Alpha and beta diversity of bacterial communities

**Figure 2:**
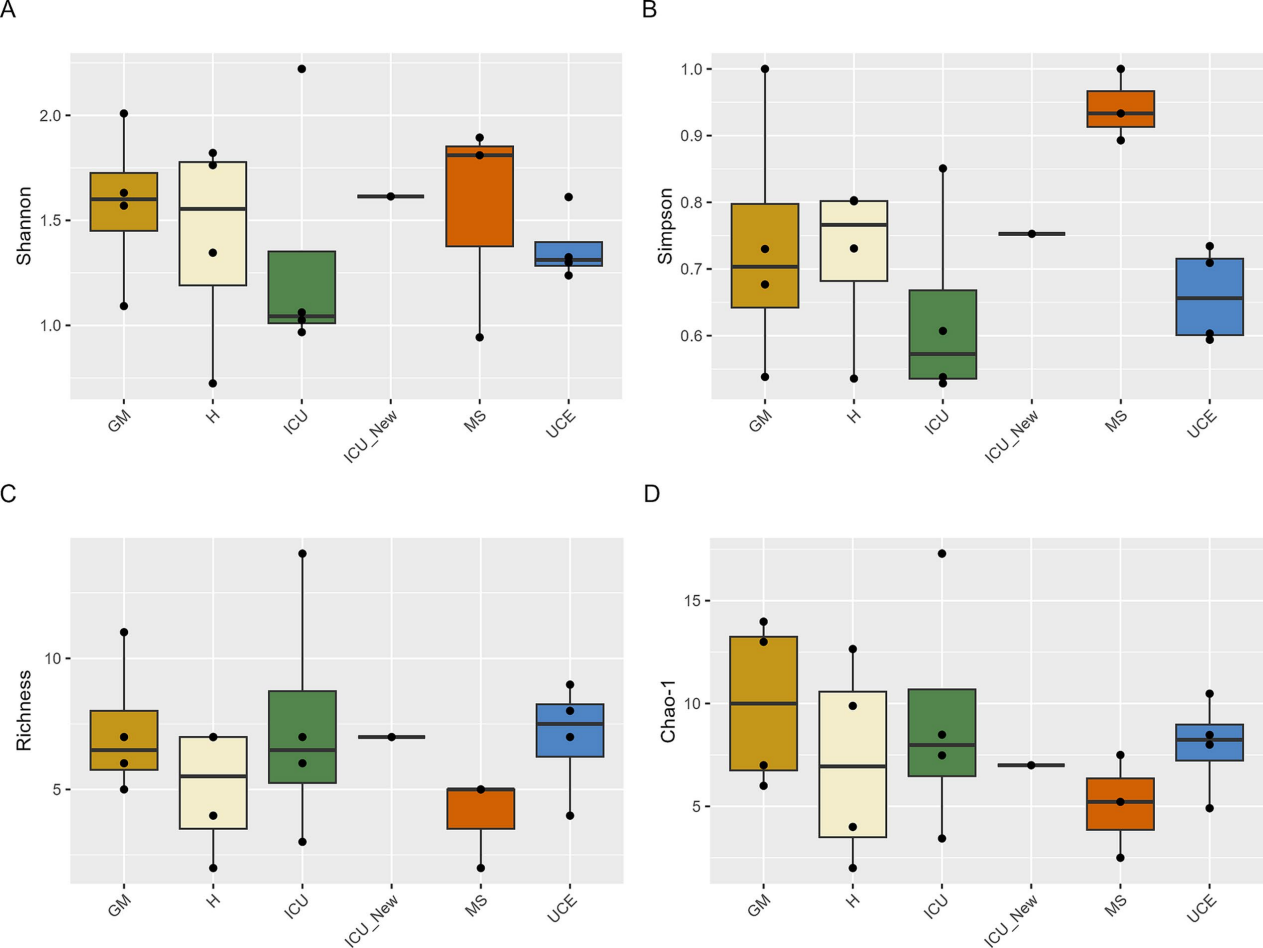
Box plot representation of Shannon **(A)**, Simpson **(B)**, Richness **(C)**, and Chao-1 **(D)** indices values for drain samples in each ward and sampling. ICU, Intensive Care Unit; MS, Microbiology Laboratory (hospital); H, Hematology; UCE, Short Stay Unit; GM, General Medicine (D3, D4, D5, D6—each drain sampling, in order). The whiskers extend to the minimum and maximum values within 1.5 times the Interquartile range (IQR) from the quartiles, with outliers indicated as individual points beyond these whiskers. Statistical analysis confirms that these differences were not significant (*p* > 0.05).

Alpha-diversity was calculated for all samplings and wards individually, showing variability across sampling with consistent patterns observed in some wards (Supplementary Table S3). Figure 2 represents the box plots for the Shannon, Simpson, Richness and Chao-1 indices for each ward. Shannon index values ranged from 0.72 (in D5 for H) to 2.22 (in D4 for ICU). Notably, D4 for ICU yielded the highest value, while D5 for H exhibited the lowest. Overall, the highest individual values are in the ICU and GM wards. Values varied across samplings, with consistency observed in GM, UCE, and ICU (excluding D4 in the ICU). Some wards exhibited an increasing trend over time, while values in MS decreased over time. H ward showed a different pattern, with values decreasing over time but increasing in D6 sampling. Additionally, the newly opened ICU in July 2022, already showed a high level of diversity. The Simpson index values were highest in the MS ward, with the others maintaining the tendency observed in the Shannon index. Richness and Chao-1, values were consistent across sampling points. None of the analyzed samples showed species dominance, with values ranging from 0 to 0.47 (Supplementary Table S3). Statistical analysis showed no significant differences between samples at a 95% confidence level.

**Figure 3:**
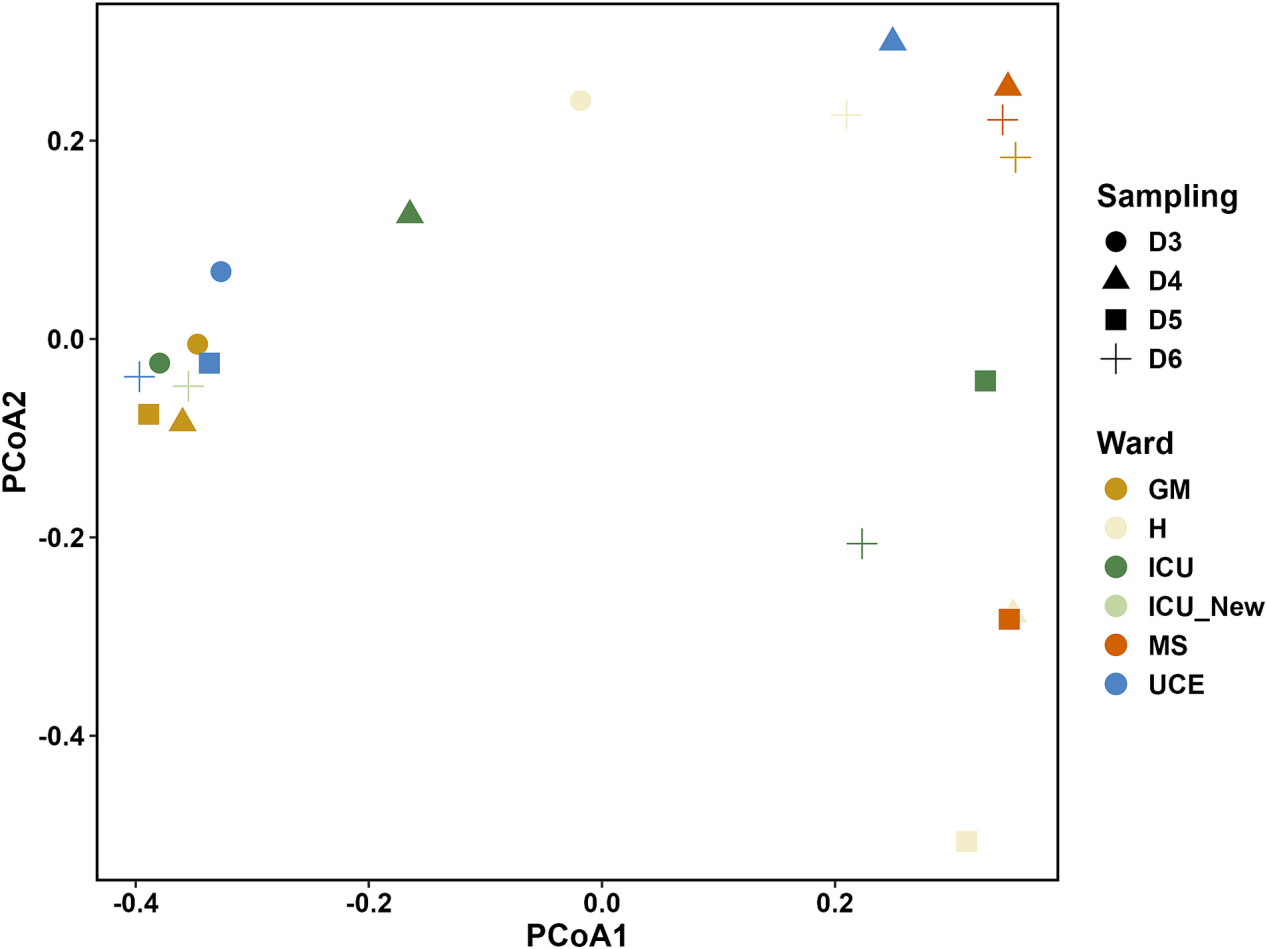
Principal Coordinate Analysis (PCoA) plot showing the relationship between the bacterial species composition for each drain sample analyzed. ICU, Intensive Care Unit; MS, Microbiology Laboratory (hospital); H, Hematology; UCE, Short Stay Unit; GM, General Medicine (D3, D4, D5, D6—each drain sampling, in order).

The PCoA plot in Figure 3 illustrates the differences in microbial communities across various wards and samplings. ICU and ICU_New wards show a more dispersed clustering pattern, particularly on D6, indicating a potential shift in the microbial community structure over time. Conversely, the UCE ward samples are closely grouped, suggesting a more stable microbial community within this ward across different samplings. Samples from the GM, H, and MS wards show varying degrees of clustering, with some overlap observed, particularly between the GM and H wards.

### 3.4 Bacterial characterization of hospital wards

**Figure 4:**
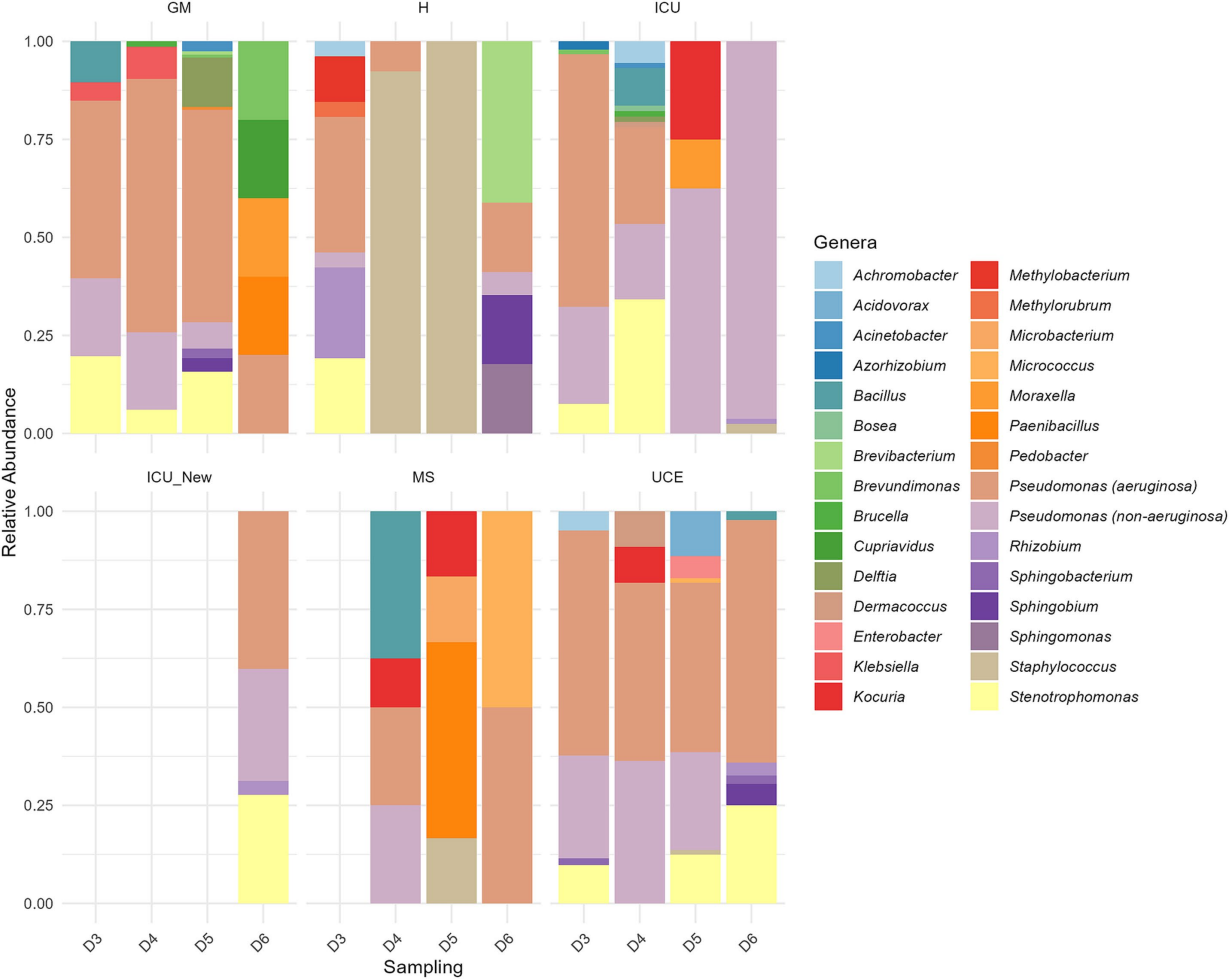
Relative abundance of each genus in the six drains analyzed in each sampling. ICU, Intensive Care Unit; MS, Microbiology Laboratory (hospital); H, Hematology; UCE, Short Stay Unit; GM, General Medicine (D3, D4, D5, D6—each drain sampling, in order). The ICU_New was only sampled once (D6) and MS had no results in D3.

The stacked bar plot in Figure 4 presents the relative abundance of microbial genera across different wards (GM, H, ICU, ICU_New, MS, UCE) and samplings (D3, D4, D5, D6). Distinct differences in microbial community composition are evident across the wards. Notably, the GM ward shows a diverse microbial profile, with a significant presence of *Pseudomonas* (both *P. aeruginosa* and non-*P. aeruginosa* species) and *Stenotrophomonas* on all sampling, excluding the last one. The H ward exhibits a highly diverse composition dominated by *Staphylococcus* in D4 and D5. In the ICU ward, a dynamic shift in microbial communities is observed over time, with non-*P. aeruginosa Pseudomonas* increasing in number and dominance over time, especially compared to a decrease in *P. aeruginosa* and *Stenotrophomonas*. Remarkably, the microbiota composition of the newly opened ICU, ICU_New, was like the two first ICU samplings (D3 and D4). The MS ward shows high variability, but with a small number of retrieved isolates. The UCE ward is characterized by a high abundance of *Pseudomonas* in general, with less variability across samplings. Seasonality had no impact on the microbial composition of evaluated sink drains.

**Figure 5:**
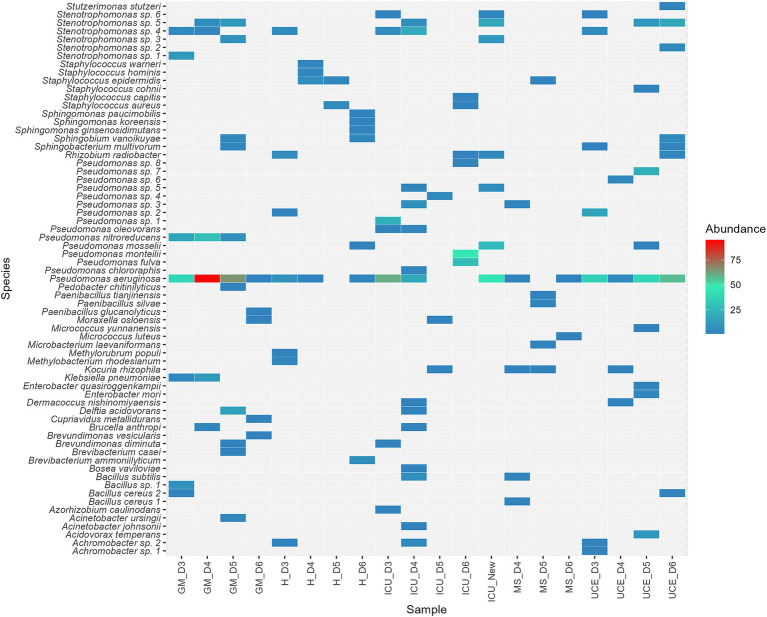
Heat map illustrating species distribution in percentage, of the 67 species detected, across all wards. Wards analyzed included: ICU, Intensive Care Unit; MS, Microbiology Laboratory; H, Hematology; UCE, Short Stay Unit; GM, General Medicine. Each letter (D3, D4, D5, D6 for drains sampling) represented a sampling point, listed in chronological order.

Figure 5 depicts the species distribution and abundance in each sampling for every ward. A total of 67 different species were identified in the sink drains. While *P. aeruginosa* stayed the most prevalent species across all wards, species composition varied in each sampling within every ward. Besides *P. aeruginosa*, at least 16 potential *Pseudomonas* species, mainly related to the *P. putida* group and six potentially different *Stenotrophomonas* species, including *S. maltophilia*, were identified. These species dominated the isolates, standing for 83% of the total number retrieved. As for the remaining species found, highlight on *K. pneumoniae* in GM*, Acinetobacter johnsonii* and *Acinetobacter ursingii* in GM and ICU, *Enterobacter mori* and *Enterobacter quasiroggenkampii* in the UCE, and *Staphylococcus aureus* in ICU and H. Furthermore, potential opportunistic pathogens such as *Achromobacter* sp.*, Brucella anthropi, Staphylococcus epidermidis* and *Stutzerimonas stutzeri* were detected.

### 3.5 Antibiotic susceptibility testing

**Figure 6:**
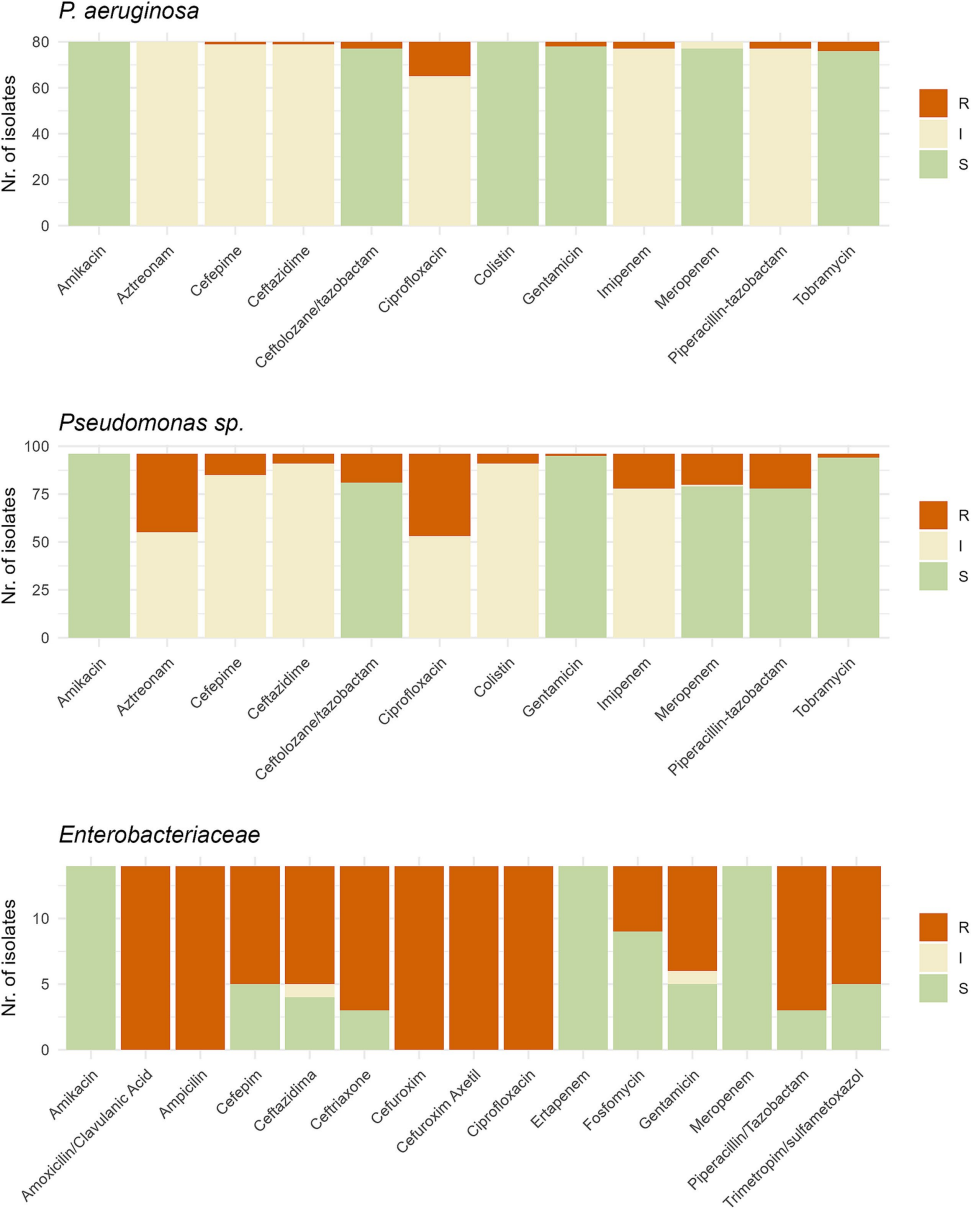
Graph representing the antibiotic susceptibility testing performed with the VITEK2 system on **(A)** 80 *Pseudomonas aeruginosa* isolates, **(B)** 93 *Pseudomonas* spp. isolates and **(C)** 14 *Enterobacteriaceae* isolates. Test was done using the 427 card for *Pseudomonas* and 425 card for *Enterobacteriaceae*. S, Susceptible; I, Intermediate; R, Resistant.

A total of 219 isolates were tested for antibiotic susceptibility using the VITEK 2 system. Testing was based on the phylogenetic assignation at species level obtained by 16S rRNA gene, with different cards used depending on the species: 425 for *Enterobacteriaceae* and 427 for *Pseudomonas* and other gram-negative species not detected on Smart CarbaID but present on ESBL agar. *Stenotrophomonas* and *Pedobacter* isolates were excluded due to intrinsic resistance and unreliable testing procedures. Total analysis time in the VITEK 2 ranged from 6–18 h. Results were categorized by *P. aeruginosa, Pseudomonas* spp. and *Enterobacteriaceae,* as depicted in Figure 6, and with more details in Supplementary Tables S4–S7. Variations in susceptibility rates were observed across different antibiotic classes and bacterial groups. Out of 80 initial *P. aeruginosa* isolates, 17 were resistant to at least one group of antibiotics (21%). Among 96 other non-*aeruginosa Pseudomonas* species, higher resistance rates were observed, with 68 isolates being resistant to at least one class of antibiotics (71%). For *Enterobacteriaceae*, *Klebsiella* and *Enterobacter* species were being resistant to third generation cephalosporins but not to carbapenems. Regarding other species, *Acinetobacter* spp. analysis provided no result for most beta-lactams tested, with *Achromobacter* and *Sphingobacterium* isolates showing resistance phenotypes to several antibiotics. All isolates tested were classified as multi-resistant (non-susceptible to three or more antibiotic classes), except for ICU_D3_83 isolate, which only had result for 4 out of 13 antibiotics tested. Twelve isolates, belonging to seven different species, yielded unreliable or no results.

Isolates resistant to ceftolozane/tazobactam, with an intermediate to resistant phenotype to carbapenems were subjected to NG-Test Carba 5 (Supplementary Table S8), with 22 isolates testing positive for *bla*_VIM_ gene presence. Gene is only found in two species, in the ICU during the first and second samplings (*Pseudomonas* sp. 1 and *P. oleovorans*), as well as in the new ICU in a different species within the *Pseudomonas* genus (*Pseudomonas* sp. 5). The only species with presence of this gene in more than one ward is *P. aeruginosa*, in which the gene was found in both the GM and UCE wards. A single isolate of *Brevundimonas diminuta* also carried the gene, in the D5 sampling of the GM ward. No *bla*_VIM_ carriers were found in H and MS wards. Other tested isolates with the NG-Test Carba 5 showed negative results to the presence of any of the 5 genes.

Notably, isolates with no results from the VITEK 2 system were included in this analysis, resulting in one *Pseudomonas* species isolates testing positive for this gene.

## 4 Discussion

Hospital environments are complex ecosystems that harbor diverse bacterial communities, including multi-resistant pathogens, which pose significant challenges for infection control (Fahy et al., 2023; Gomila et al., 2005; Sukhum et al., 2022). Sink drains, identified as critical reservoirs, facilitate bacterial colonization due to frequent contact with patients and healthcare workers (Volling et al., 2020). This study provides a comprehensive investigation of bacterial diversity across various hospital wards over one-year, emphasizing the importance of understanding these dynamics for effective infection control strategies. The findings highlight varying bacterial counts across different media, challenges in species identification, and the implications of antibiotic resistance. The discussion explores temporal changes, recolonization patterns, and the prevalence of antibiotic-resistant bacteria, underscoring the need for continuous monitoring and enhanced cleaning protocols to mitigate infection risks in healthcare settings.

Previous research has primarily focused on known pathogens in the ICU or used culture-independent approaches that do not allow for bacterial isolation (Cronquist et al., 2012). While this study is limited to one hospital, it adopts a comprehensive approach by examining bacterial diversity across various wards, including recognized pathogens and potential emerging threats, not just at a specific moment, but over the course of a year. This approach allows for meaningful comparisons between wards, although generalization of results to other hospital settings may be challenging.

The findings indicate higher CFU/mL counts on room temperature plates compared to 37°C, suggesting that these conditions and extended incubation times (ranging from 72 h to 7 days) facilitated the detection of broader range of bacterial groups (Gomila et al., 2005; Pirzadian et al., 2020). Furthermore, most of the retrieved bacteria are environmental bacteria, which are naturally adapted to cooler temperatures, such as *Pseudomonas* or *Acinetobacter* in hospitals. Biofilm formation by these bacteria also favors growth at lower temperatures and disinfectant resistance (Bridier et al., 2011). The presence of bacteria that usually do not grow at 37°C, but some do grow at higher temperatures and can potentially be pathogenic like *Legionella* spp. in water systems, which is difficult to culture at 37°C, but is a risk to immunocompromised patients nonetheless (Moritz et al., 2010).

This study highlights how the combination of different culture media, both non-selective and antibiotic-supplemented, provides a comprehensive and complementary view of the bacteria present (Supplementary Tables S9, S10). R2A agar, which yielded the highest number of unique species and an evenly distributed number of isolates, proved to be a valuable indicator for such studies. Notably, ESBL media revealed growth comparable to R2A in the new ICU, likely due to bacteria like *Pseudomonas* and *Stenotrophomonas* thriving in both media types, as well as environmentally. Antibiotic-supplemented media highlighted the growth of non-traditional pathogenic species, particularly within the *Pseudomonas putida* group, suggesting these bacteria could serve as potential reservoirs for resistance genes (Brovedan et al., 2021; Meireles et al., 2013; Mulet et al., 2013).

Despite selecting up to five isolates of each unique morphology in each plate and culture media, species with similar morphologies may have been missed or overrepresented, such as *P. aeruginosa*. The challenge of non-cultivable or difficult-to-culture bacteria remains, although longer incubation times may have facilitated the growth and isolation of some.

The combination of MALDI-TOF MS and 16S rRNA gene sequencing enabled comprehensive characterization and species definition. Sequencing of this gene revealed a potential novel species within the *Paenibacillus* genus, with a consensus sequence below the 98.7% identity threshold typically used to define new species (Caudill and Brayton, 2022). However, species-level identification was challenging for *Pseudomonas putida*-related species and *Stenotrophomonas* spp. due to similar protein profiles and 16S rRNA genes, indicating the need for further studies. For these genera, sequencing housekeeping genes like *rpoD* for *Pseudomonas* species and *gyrB* for *Stenotrophomonas*, or whole-genome sequencing (WGS) may offer more precise identification (Mulet et al., 2009; Svensson-Stadler et al., 2012).

Diversity indexes were used to assess microbial composition across wards. Alpha-diversity values included isolates from all culture media to ensure representation of all detected species. Although selective media might bias results, this approach provided a thorough overview of all represented species. The highest individual Shannon and Simpson indexes were observed in the ICU and GM wards, while overall highest values are in GM, MS and H. It is important to highlight that the MS ward had very few isolates retrieved, but all represented different species, which may have influenced these results. Nevertheless, these high diversity values can be troublesome as it suggests a higher risk of pathogen presence in these wards with immunocompromised patients. The new ICU exhibited high diversity values similar to the old ICU, indicating rapid bacterial colonization, as previously reported in a study by Lax et al. (2017). The similarity in Richness and Chao-1 indexes across sampling points suggests that our results align with true richness expectations reported in metagenomic studies (Chen et al., 2017; Shobo et al., 2020). Dominance values showed that while *P. aeruginosa* was prevalent, it did not dominate the population.

The results suggest that the composition of culturable microbiota in drains evolves over time, with *Pseudomonas* and *Stenotrophomonas* remaining predominant genera, though varying in species. This is likely due to factors such as cleaning protocols, ward utilization and antibiotic usage (Jung et al., 2019). As previous findings suggest, while long-term colonization of sink drains seems difficult, recolonization is effective and generally involves bacteria from external sources, particularly in less urban hospital settings (Suleyman et al., 2018). These dynamic underscores the importance of continuous monitoring and improved cleaning protocols to limit bacterial growth (Klassert et al., 2022). Interestingly, many of the bacterial taxa identified were associated with plants, such as *Pseudomonas, Methylobacterium*, and *Rhizobium* (Oberauner et al., 2013). Furthermore, a relationship between ward usage and *P. aeruginosa* presence was found, in line with previous studies (Crone et al., 2020). Notably, CFU/mL was absent or present at low counts in the analyzed MS samples. These samples were collected from one of the microbiology unit drains, which were frequently cleaned with disinfectants such as bleach. Antibiotic resistance is a significant public health threat in healthcare settings (D’Souza et al., 2019). Our antibiotic susceptibility testing revealed several multi-resistant isolates, which includes *Klebsiella, Enterobacter* species, and *P. aeruginosa*. ESKAPE pathogens found in our study exhibit alarming resistance levels, often associated with higher virulence (Geisinger and Isberg, 2017; Schroeder et al., 2017). The WHO priority pathogens list includes several bacteria identified in our study, emphasizing the urgent need for new antibiotics and surveillance (WHO, 2024).

Notably, most *Pseudomonas* isolates were resistant to at least one carbapenem, particularly among the *P. putida* groups species, highlighting the potential infections from these non-typical pathogenic species (Mulet et al., 2017). The NG-Carba test detected *bla*_VIM_ genes in 22 isolates across several species, predominantly in the ICU, indicating a need for broader screening beyond traditional 37°C incubation, as one of these species was not culturable above 30°C. These results suggest that selective pressure in hospitals caused by antibiotic overuse also plays a role in selecting bacteria that thrive at lower incubation temperatures. Two of the species found in the ICU (*Pseudomonas* sp. 1 and *Pseudomonas* sp. 5) belong to the *P. putida* group, reinforcing that these bacteria may indeed be carriers of antibiotic resistance genes, given that this gene is often associated to a class 1 integron, embedded in transposons and plasmids (Zhao and Hu, 2011). Regarding *P. aeruginosa*, the *bla*_VIM_ gene was found in isolates from the UCE and GM wards during the D6 sampling. Despite the low number of total isolates in the GM ward, the detection of a *bla*_VIM_-positive *P. aeruginosa* isolate is noteworthy. This finding suggests a possible connection between these isolates, indicating either the spread of bacteria between wards, or a common source of contamination. However, further studies are needed to confirm these findings.

The presence of *bla*_VIM_ in a *Brevundimonas* isolate, though rare, highlights the evolving landscape of antibiotic resistance, with this work being one of the few reports of this gene in the species (Almuzara et al., 2012; Altamirano-Beltrán et al., 2023; Scotta et al., 2011).

Broader sampling across multiple hospitals and regions is crucial to obtain a comprehensive understanding of bacterial diversity and resistance patterns. This should include simultaneous environmental and clinical sampling to link environmental contamination with patient infections, as well as temporal sampling to track changes over time considering factors like seasonality and hospital practices.

Although this study does not include a direct comparison between our isolates and clinical isolates, further investigation would be valuable to assess the clinical relevance of environmental bacteria. Genotypic and phenotypic comparisons can help identify potential reservoirs of nosocomial infections and help to understand resistance gene transfer. A direct relationship has previously been reported between the presence of antibiotic resistant bacteria in sink drains and hospital infections, particularly in ICU, suggesting that the bacteria found in our study may have contributed to infections, specially through aerosol transmission (Döring et al., 1991; Wolf et al., 2014). A recent study by Volling et al. (2024), revealed that 7% of *P. aeruginosa* from sink drains were associated with nosocomial infections, underscoring the importance of such comparisons.

## 5 Conclusion

Our findings reveal that hospital sink drains can act as reservoirs for both known and emerging pathogens, exhibiting and alarming presence of antibiotic resistance genes. The use of culturomics should be more widely considered, as it facilitates bacterial isolation, enabling better species discrimination and supporting further research. Additionally, it allows for the visualization of colonization dynamics, as observed in this study, which can be challenging to visualize through metagenomics alone. This study also highlights the need for improved infection control practices. Developing and implementing enhanced cleaning protocols, particularly for high-risk areas like sink drains, is critical. Routine microbial monitoring and targeted disinfection strategies should be employed to address multi-resistant bacteria. New strategies, such as antibiotic stewardship programs, novel therapeutics (like bacteriophage therapy), and ongoing education for healthcare workers are essential to control bacterial contamination. Policy and regulatory measures should standardize and update infection control practices, with increased collaboration and data sharing to improve patient outcomes and public health.

By addressing these needs and implementing these measures, healthcare facilities can better manage bacterial contamination and reduce the incidence of hospital-acquired infections.

## Data Availability

The original contributions presented in the study are included in the article/supplementary material. The R scripts used for all images and statistical analysis for this study can be found in the DrainsDiversityArticle repository (https://github.com/zelaco/DrainsDiversityArticle). Further inquiries can be directed to the corresponding author.
